# Genetic and Epigenetic Regulation of Brain Organoids

**DOI:** 10.3389/fcell.2022.948818

**Published:** 2022-07-01

**Authors:** You-Wei Wang, Nan Hu, Xiao-Hong Li

**Affiliations:** Academy of Medical Engineering and Translational Medicine, Tianjin University, Tianjin, China

**Keywords:** brain organoids, gene mutation, epigenetics, autistic spectrum disorders, genetics

## Abstract

Revealing the mechanisms of neural development and the pathogenesis of neural diseases are one of the most challenging missions in life science. Pluripotent stem cells derived brain organoids mimic the development, maturation, signal generation, and function of human brains, providing unique advantage for neurology. Single-cell RNA sequencing (scRNA-Seq) and multielectrode array independently revealed the similarity between brain organoids and immature human brain at early developmental stages, in the context of gene transcription and dynamic network of neuronal signals. Brain organoids provided the unique opportunity to investigate the underlying mechanism of neural differentiation, senescence, and pathogenesis. In this review, we summarized the latest knowledge and technology in the brain organoid field, the current and potential applications in disease models and pre-clinic studies, with emphasizing the importance of transcriptional and epigenetic analysis.

## Introduction

**FIGURE 1:**
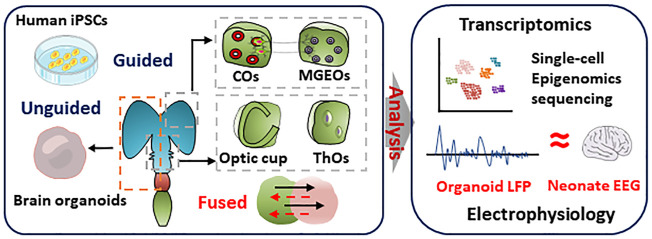
Genetic and epigenetic regulation in the brain organoids. Top, genetics in brain organoids. iPSCs carrying ASD or SCZ pathogenic genes are derived from patients or gene editing. The mutation of ASD or SCZ are referring to the commonly mutated genes in GWAS databases, which are shown in middle. The ASD organoids showed macrocephaly, microcephaly phenotypes, and over-produced GABAergic neurons. The SCZ-organoids characterized abnormal proliferation of progenitor, reduced mature neurons, and disruption of synaptic function. Bottom, epigenetics in brain organoids. Combined with single-cell sequencing, ATACseq and CHIP-seq analyze epigenetics during brain organoids and human brain development, including DNA methylation, histone variants, non-coding RNA, and chromatin accessibility. Epigenetics can regulate timing during development. Epigenetic regulation is deeply influenced by environmental factors. Exposure to toxic substances, viruses, alcohol, and stress may have epigenetic effects on fetal brain development. Brain organoids infected with Zika virus during early development showed epigenetic abnormalities, impaired progenitor proliferation as well as a distinct microcephaly phenotype.

The human brain uses 100 billion neurons and 100 trillion connections between neurons to receive signals, generates consciousness, and sends orders to target organs, which makes it the almost perfect information processing system. All of the neurons derived from neural stem cells. The development of human brain is dependent on the information encoded in genomic sequence and regulated by epigenetic modification (Barkovich et al., 2012). Because of the critical ethical concerns, there are lots of untouchable field in the molecular mechanisms of human brain. Brain organoids can mimic the cell composition, hierarchical structure, electronical network, and disorders of human brain. From the perspective of transcriptome and epigenome atlas, significant similarity was observed between iPSCs (induced pluripotent stem cells) derived brain organoids and human brain (Amiri et al., 2018) (Figure 1). Reintroduction of a mutated gene in brain organoids provides not only the opportunity to study the function of a specific gene in the context of neural development, but also valuable disease models for neural diseases, which includes autism spectrum disorders, schizophrenia, and others.

## Genetic Factors of Neural Development and Diseases: from Human Brain to Brain Organoids

### Brain Organoids Provide Unique Advantage in Hominin Evolution Studies

There are many hominin lineage branches in the evolution path of humans. They have similar but slightly different genomic sequences. The fossil record provides the genetic sequences of Neanderthals and Denisovans (Green et al., 2010; Meyer et al., 2012). An interesting idea is investigating the contribution of genetic sequence difference in human brain evolution among different hominin lineages. It is not possible to directly compare the living cells and tissue between hominins and humans. Using iPSCs and CRISPR-Cas9 technology, hominin genes were introduced into human cells. Brain organoids were generated from iPSCs possess hominin genes. The differences in synaptogenesis, neural network, and electrophysical traits are observed between brain organoids with modern and archaic genes, which provide critical evidence to study the evolution of humans (Trujillo et al., 2021).

### Brain Organoids Reveal the Genetic Mechanism of Neural Diseases

Brain organoids were used as disease models in multiple neural disorders, which are mainly caused by abnormal neural cell survival, proliferation, and differentiation. This is reviewed in some other reviews (Sidhaye and Knoblich 2021). For emphasizing the advantage of brain organoids in stem cell biology and neurology, we choose autism spectrum disorder (ASD) and schizophrenia to illustrate the unique role brain organoids played in revealing the pathogenesis mechanisms and discovering potential therapeutic strategies. Based on the genetic information, cortex structural, and functional regions, the correlation between DNA sequences and cortical surface area was established (Chen et al., 2012), which emphasizes the importance of genome in human brain development and function. Taking the advantage of whole gene sequencing, increasing disease-associated genes were identified.

**FIGURE 2:**
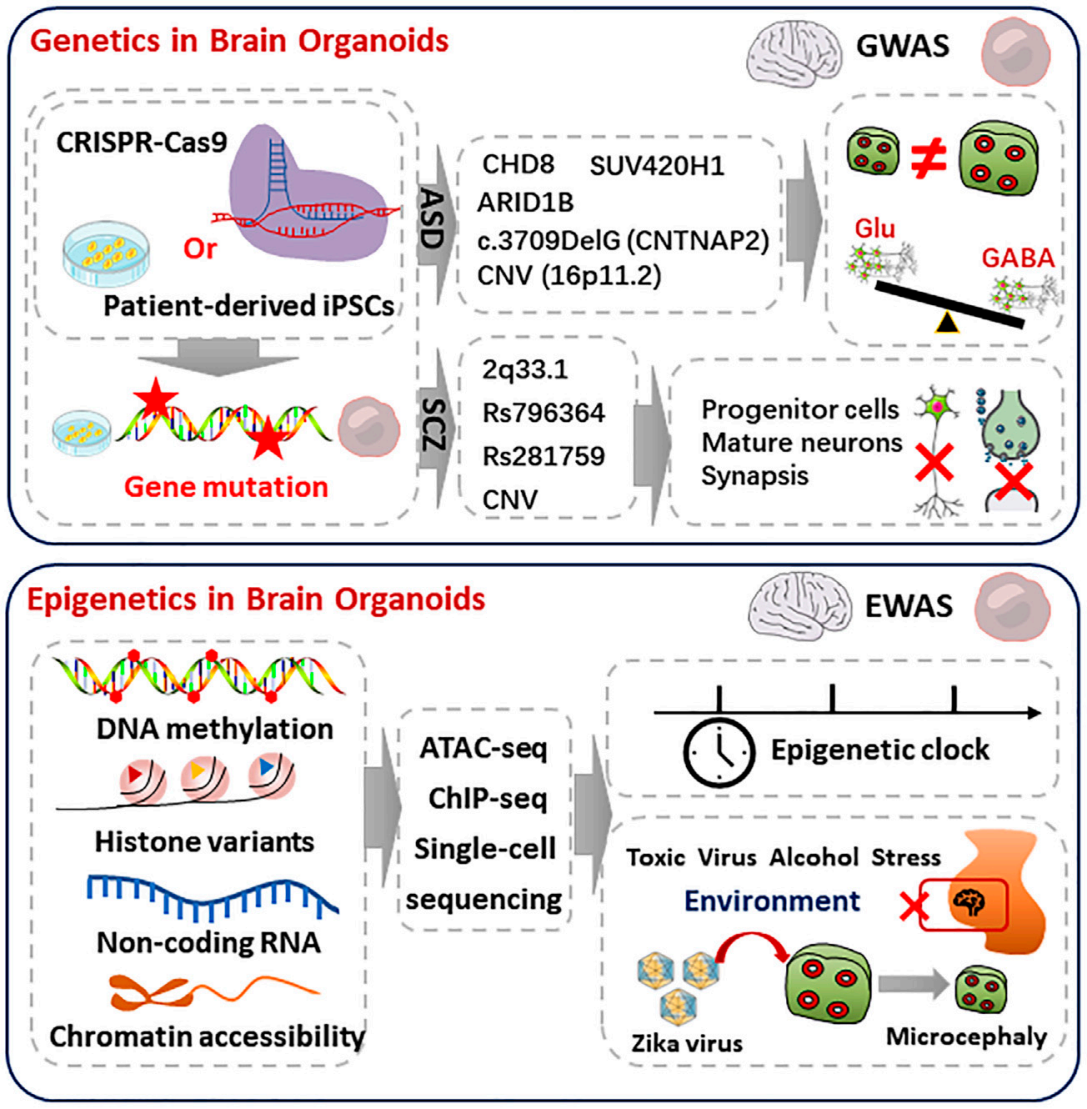
Reality of brain organoids in simulating human brain. Brain organoids and region-specific brain organoids were induced by undirected or directed differentiation, respectively. Single-cell sequencing and transcriptome analysis was performed to characterize the similarity of cellular composition and gene expression in brain organoids and human brain. Multi-electrode arrays detected the electrophysiological signals of cortical brain organoids that is similar to EEG signals in preterm infants

The mutation of some genes was considered to contribute to the initiation of ASD, which includes but not limited to CAPRIN1, AFF2(Jiang et al., 2013). Another different kind of genetic change, copy number variation, was also linked to ASD (Levy et al., 2011) (Figure 2). Investigation revealed that deletion and duplication of genes have less impact on females than males. More researches are needed to answer if environmental factors played a role in the unequal frequency between different genders.

Besides mutation in the coding regions, more ASD-associated changes were observed in the non-coding region of genome. These regions not only include expression regulating factors, but also hold a number of regulations of splicing differences (Walker et al., 2019). Genetic sequencing of patient samples established the correlation between genes and neural diseases. However, it is hard to reveal the mechanisms by which the roles of disease-causing genes played in regulating neuron function and neural network, based on the data directly derived from patient samples. Based on scRNA-seq (Single-cell RNA sequencing) analysis, remarkable similarity was observed between *in vitro* human brain organoids and human fetal cortical tissues. Using the transcriptome-based unbiased clustering, neural progenitor cells and diverse differentiated cells were identified in brain organoids, which recapitulates the hierarchies, organization, and developmental trajectories of human fetal neocortex (Camp et al., 2015). However, no technique or evidence is available to investigate whether advanced neural functions exist in brain organoids.

Impairments in social communication and behavior patterns are the main clinical signs of ASD. The abnormal development of neural system of ASD starts from embryonic stage, which usually shows symptoms in childhood. Although genetic mutations are considered to be the principle causing of ASD, only 10% ASD patients were identified by known genetic condition. More investigations are needed to establish the clear linkage between inherited material and ASD. Thanks to stem cell and genome editing technology, patient-derived brain organoids provided the unique opportunity to investigate the mechanisms by which disease-driving genes impair the neurons. By comparing organoids derived from iPSCs of autism patients and their unaffected family members, differences in cell proliferation, synaptogenesis, and GABA/glutamate neuron differentiation were observed, although there is no significant neuronal organization and excitability. To explore the mechanism by which genes with different expression contribute to the ASD-associated phenotypes, FOXG1 was selected and its expression in brain organoids was regulated by lentiviruses carrying short hairpin RNAs, which is almost not possible in patients or patient-derived tissues. Attenuated FOXG1 expression restored the GABAergic neuronal differentiation to the normal level. These data revealed that FOXG1 might be an important driver of ASD neural phenotypes (Mariani et al., 2015).

Homozygous loss-of-function mutation in contactin-associated-protein-like 2 (*CNTNAP2*) causes a special kind of ASD, with the symptoms of early-onset epilepsy and increased head circumference (Strauss et al., 2006). Although knockouting Caspr2 causes some neural dysfunction, it also misses some critical disease-related symptoms, such as hyperproliferation of neural cells (Poliak et al., 2003). Increased proliferation and total number were observed in the brain organoids derived from ASD patients who harbored CNTNAP2 mutation, which provided the possible mechanism of increased head circumference of the patients. ScRNA-seq of organoids revealed that CNTNAP2 dominantly expressed in PFC-excitatory neurons and the targets genes regulated by CNTNAP2 mainly enriched in ASD-associated genes (de Jong et al., 2021). These studies emphasized the unique advantage of brain organoids in recapturing the phenotypes which are exclusively present in human neural system. Organoids helped neurologists uncover the role that inherited material plays in ASD. However, there is few investigations focusing on gene-environment interactions. More studies or new methods are needed to explore deeper mechanism of ASD from environmental factors perspectives.

One percent of human population suffers from schizophrenia, which is a chronic mental disorder with major signs of psychosis and cognitive impairment (Plooster et al., 2022). Most of the schizophrenia associated loci are protein-coding genes, which include involved not only glutamatergic neurotransmission and synaptic plasticity gene, but also the target of antipsychotic drugs. Besides brain tissue, schizophrenia linked genome association enriched in B lymphocytes as well, the antibody-producing cells (Schizophrenia Working Group of the Psychiatric Genomics 2014). This observation might explain the relationship between autoimmune diseases and schizophrenia, and the mechanism of some self-antibodies were found in schizophrenia patients with much higher proportion than healthy individuals (Benros et al., 2012).

Impaired neural differentiation and disrupted neuron quantity were observed in schizophrenia patient-derived organoids, compared with the organoids derived from healthy donors. Compared to organoids derived from healthy donors, there are more apoptosis, neural progenitors, in the ventricular zone of schizophrenia patients derived organoids (Notaras et al., 2021a). This is one of the unique advantages of 3D organoids, as brain structures usually do not exist in 2D *in vitro* neural culture.

Schizophrenia-derived iPSCs and the derived brain organoids provide novel *in vitro* model to investigate the psychiatric disorder from the perspective of etiology, mechanism, and therapy. Hundreds of different expression genes between schizophrenia patients and healthy donors derived organoids were identified by total RNA sequencing. Almost quarter of these genes are overlapped with schizophrenia associated genes discovered by GWAS (Kathuria et al., 2020). They enriched in neurodevelopment, metabolism, neural function, and immune responses. Although the genes which differently expressed in schizophrenia organoids enriched in immune response as well, few researches investigated the brain organoids from immunological perspectives. Schizophrenia organoids were also subjected to quantitative proteomic analysis. Similar defects were observed in axon and neuronal differentiation at protein level (Notaras et al., 2021b), which is consistent with transcriptome and genome researches.

### Epigenetic Modification of Human Brain and Brain Organoids

Epigenome is a reversible change of inherited materials. Epigenetic change does not alter a DNA sequence. It changes and tunes the transcription of DNA sequence. Epigenomics mainly encompasses DNA methylation, histone modification, and noncoding RNA mediated transcriptional regulation. Environment, age, behavior, and diseases can cause epigenetic changes (Figure 2). Epigenetic regulation plays a critical role in tissue development, homeostasis, pathogenesis, and senescence (Vezzani et al., 2022).

On top of protein-coding genes, non-coding regions of human genome are also found to be critical in human cortical neurogenesis (de la Torre-Ubieta et al., 2018).

Epigenetic and gene expression between human and non-human primate brains are extremely distinct due to evolution of species, which are expected to interpret the epigenetic changes in human-specific sequences (Mendizabal et al., 2016). The interplay of the activation and inhibition of multiple developmental signaling pathways in brain development was tightly controlled by epigenetics (Imamura et al., 2014). Whole-genome single-base analysis revealed that dynamic genome non-CG methylation occurred in the brain development of fetal to adult, which characterized constant changes at different developmental stages and specifically accumulated in neurons or glial cells, respectively (Lister et al., 2013). The cell-type-specific 3D epigenomes revealed new laws of cis-acting element regulation of gene expression during cell fate determination, was expected to provide new reference data for brain development and neuropsychiatric disorders (Song et al., 2020). The epigenetics of iPSC-derived neurons has been extensively studied, which was limited by the lack of complex networks (Vieira et al., 2019).

Based on histone mark (methylation and acetylation) ChIP-seq brain organoids at different developmental stages, dynamic epigenetic traits were observed along with organoids maturation. After compared with brain tissue data from the PsychENCODE developmental dataset, similar enhancer active models were observed between brain organoids and human brain development. This implied that brain organoids might be a good tool to recapitulate neural system development. As epigenetic modification is considered as reversible, brain organoids are also a valuable platform to investigate and evaluate potential therapies.

Epigenome-wide association studies (EWAS) (Verma, 2016) is an important method to study the variation in epigenetic level, mainly DNA methylation. In a study focusing on AD, EWAS revealed that ankyrin 1 (ANK1) is one of the AD-associated genes with abnormal epigenetic modification. The hypermethylation of ANK1 was observed in AD manifested region of brain, the entorhinal cortex, not in the regions which were protected from AD-related neurodegeneration (cerebellum), nor peripheral blood (Lunnon et al., 2014). Increased evidence suggested that epigenetic change played an important role in the development of schizophrenia. However, genetic studies dominate this niche, outnumbering epigenetic studies. More investigation, especially fundamental basic studies, about the contribution of epigenetic factors is needed to clarify the development of schizophrenia and find potential interventions for mental illness (Francisco et al., 2022). Brain organoid recaptured early non-CG methylation (an epigenetic modification suppressing gene expression) of super-enhancers and demethylation profiles of fatal human brain (Luo et al., 2016). This supports the possibility of using organoids to model epigenetic change in human brain development and neural disorders.

The opening or closing of genes anomalously controlled by epigenetics could lead to abnormal gene expression and neurodevelopmental disorders. Especially, environmental factors cannot be ignored. Prenatal exposure to risk factors such as alcohol, heavy metals, toxic substances, as well as maternal immune activation may induce epigenetic changes (Portales-Casamar et al., 2016). Epigenetic changes in brain organoids infected with the Zika virus were analyzed by whole-genome bisulfite sequencing (WGBS) beyond ethical and species-specific limitations. The results showed that Zika virus was able to alter DNA methylation at specific gene loci and infected-brain organoids displayed a microcephaly phenotype (Janssens et al., 2018). Epigenetic editing regulates target gene expression from the transcription without modification of gene sequences (Holtzman and Gersbach 2018). Epigenetic alterations have a potential of reshaping the mechanism between chromatin state, gene regulation, and cellular phenotype (Jurkowski et al., 2015). Epigenetic editing based on brain organoids is expected to reveal disease mechanisms and treatment techniques *in vitro*. Based on CRISPR-cas9 system, precise genetic editing was achieved and was used in brain organoids to investigating gene function in neural development and their contribution in neural diseases. However, epigenetic editing tools usually act globally. Site specific epigenetic editing may not only improve current understanding about the role of epigenetic marks played in neural diseases, but also provide novel opportunity to develop next generation therapeutic strategies targeting epigenetic factors.

## Conclusion

Brain organoid shows extraordinary potential in neurology. The differentiation of stem cells in brain organoids is still a self-organization process. Although different brain regions are generated by regionally specific culture system, many human brain structures do not exist in brain organoids. Few neural development and diseases can be recapitulated in brain organoids. Revolution or professional cell culture devices are needed to improve the cellular and structure diversity of brain organoids.

Functional maturation of a brain organoid needs more than 3 months. Reliable electrical signals usually merge arise 6-month differentiation. This extremely long *in vitro* culture makes brain organoids to be vulnerable to contamination of infection and external environment. It also increases the organoid-to-organoid variability, which seriously impaired the comparability of data generated from different laboratories. Although significant similarity was observed between human brain and brain organoids, there is still nonnegligible brain-organoids variation, in the perspective of electrical function, cellular diversity, and structural integrity. Electrical activity is one of the most critical neural functions of brain organoids. Traditional MEA can not measure the inside electrical signal of 3D brain organoids. Although few studies use 3D electrodes analyzing the deep signal of brain organoids, the number of electrodes is much smaller than MEA. Besides transcriptomic similarity, brain organoids show action potentials and electrophysiological responses, the signature characteristics of human brain neural network (Logan et al., 2020). Some computational toolkits, such as VoxHunt, were developed to anchor the organoid data to brain references, which accelerated and significantly improve the accuracy in organoids analysis (Fleck et al., 2021). Although there are still some technical obstacles. Brain organoids provide a unique and unreplaceable opportunity to build up, investigate, and redesign the neuron, the neural diseases, and the neural system. It will also be a benefit to developing novel therapeutic strategies.

## Abbreviations

COs, cortical brain organoids; MGEOs, medial ganglion protrusion brain organoids; ThOs, thalamic organoids; iPSCs, induced pluripotent stem cells; GWAS, Genome-wide association study; EWAS, epigenome-wide association studies; ASD, autistic spectrum disorders; SCZ, schizophrenia; CNV, Copy number variations; ATAC-seq, Assay for Transposase-Accessible Chromatin with high throughput sequencing; CHIP-seq, Chromatin Immunoprecipitation Sequencing
